# Long-term impact of maternal obesity on the gliovascular unit and ephrin signaling in the hippocampus of adult offspring

**DOI:** 10.1186/s12974-024-03030-w

**Published:** 2024-02-02

**Authors:** Seyedeh Marziyeh Jabbari Shiadeh, Fanny Goretta, Pernilla Svedin, Thomas Jansson, Carina Mallard, Maryam Ardalan

**Affiliations:** 1 Department of Physiology, Institute of Neuroscience and Physiology, Sahlgrenska Academy, University of Gothenburg, Gothenburg, Sweden; 2 Department of Clinical Medicine, Translational Neuropsychiatry Unit, Aarhus University, Aarhus, Denmark; 3 Division of Reproductive Sciences, Department of OB/GYN, University of Colorado, Anschutz Medical Campus, Aurora, CO USA

**Keywords:** Fetal programming, Brain plasticity, Obesity in pregnancy

## Abstract

**Background:**

Children born to obese mothers are at increased risk of developing mood disorders and cognitive impairment. Experimental studies have reported structural changes in the brain such as the gliovascular unit as well as activation of neuroinflammatory cells as a part of neuroinflammation processing in aged offspring of obese mothers. However, the molecular mechanisms linking maternal obesity to poor neurodevelopmental outcomes are not well established. The ephrin system plays a major role in a variety of cellular processes including cell–cell interaction, synaptic plasticity, and long-term potentiation. Therefore, in this study we determined the impact of maternal obesity in pregnancy on cortical, hippocampal development, vasculature and ephrin-A3/EphA4-signaling, in the adult offspring in mice.

**Methods:**

Maternal obesity was induced in mice by a high fat/high sugar Western type of diet (HF/HS). We collected brain tissue (prefrontal cortex and hippocampus) from 6-month-old offspring of obese and lean (control) dams. Hippocampal volume, cortical thickness, myelination of white matter, density of astrocytes and microglia in relation to their activity were analyzed using 3-D stereological quantification. mRNA expression of ephrin-A3, EphA4 and synaptic markers were measured by qPCR in the brain tissue. Moreover, expression of gap junction protein connexin-43, lipocalin-2, and vascular CD31/Aquaporin 4 were determined in the hippocampus by immunohistochemistry.

**Results:**

Volume of hippocampus and cortical thickness were significantly smaller, and myelination impaired, while mRNA levels of hippocampal EphA4 and post-synaptic density (PSD) 95 were significantly lower in the hippocampus in the offspring of obese dams as compared to offspring of controls. Further analysis of the hippocampal gliovascular unit indicated higher coverage of capillaries by astrocytic end-feet, expression of connexin-43 and lipocalin-2 in endothelial cells in the offspring of obese dams. In addition, offspring of obese dams demonstrated activation of microglia together with higher density of cells, while astrocyte cell density was lower.

**Conclusion:**

Maternal obesity affects brain size, impairs myelination, disrupts the hippocampal gliovascular unit and decreases the mRNA expression of EphA4 and PSD-95 in the hippocampus of adult offspring. These results indicate that the vasculature–glia cross-talk may be an important mediator of altered synaptic plasticity, which could be a link between maternal obesity and neurodevelopmental/neuropsychiatric disorders in the offspring.

**Graphical Abstract:**

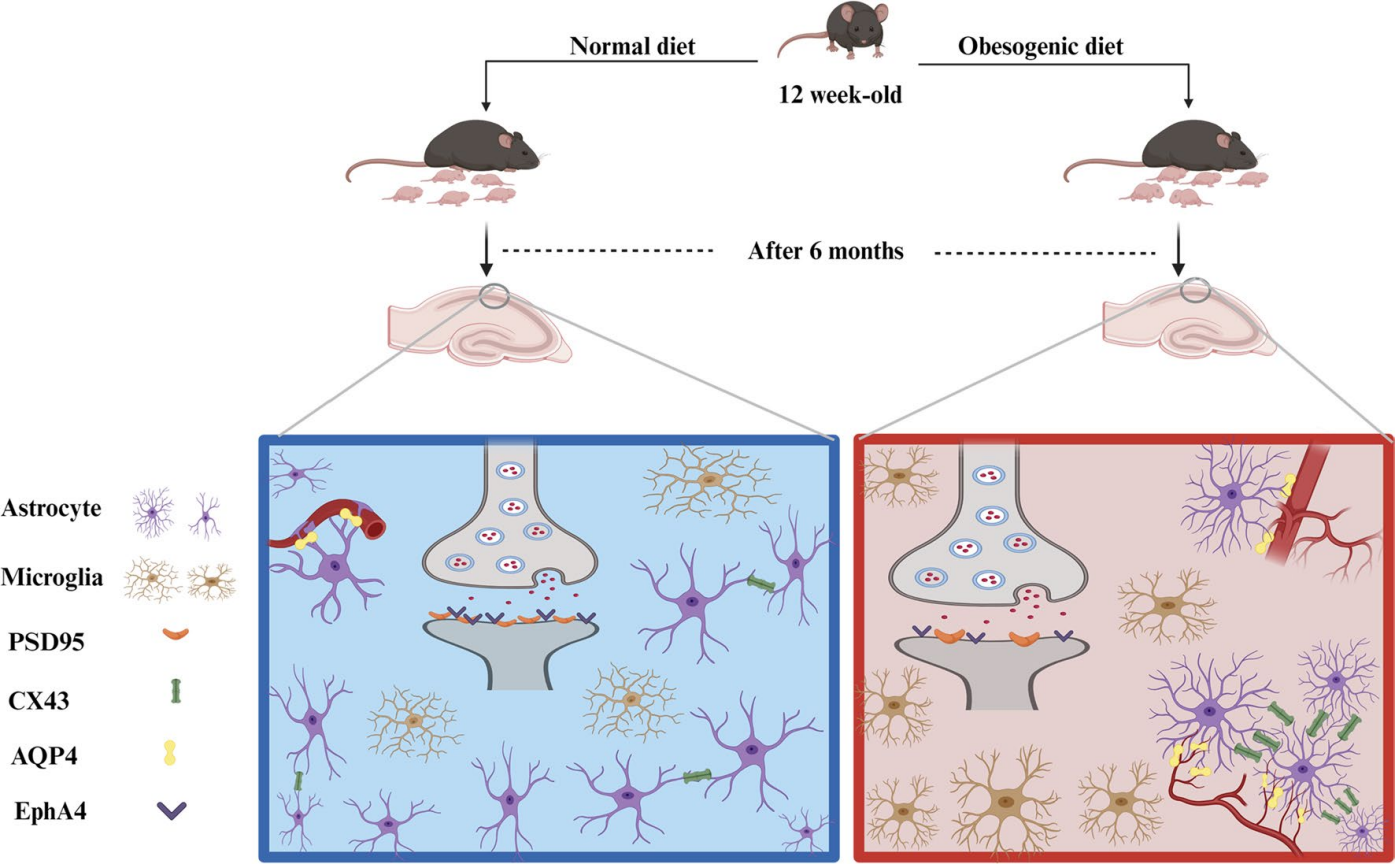

## Introduction

Maternal obesity in pregnancy influences the health of both mother and child [1, 2]. For example, offspring of obese mothers has an increased risk of developing metabolic diseases such as insulin resistance and type 2 diabetes [3, 4]. The fetal and neonatal brain undergo rapid changes during development and environmental factors such as inflammation and nutritional changes have impact on the central nervous system [5, 6]. High maternal body mass index (BMI) has also been linked to neurodevelopmental morbidity in children, including lower cognitive performance and intellectual disability [7]. Significant evidence in human cohort studies points towards an increase in neuropsychiatric disorders in children born to obese mothers [8, 9]. Maternal obesity is linked to modifications in the inflammatory profiles of the mother, fetus, and placenta. These alterations can program changes in organogenesis, tissue development, and metabolism, ultimately predisposing offspring to adult disease [10]^10^. Animal experimental studies have shown that maternal obesity impair cognitive function in association with reduced dendritic arborization of hippocampal neurons in young offspring [12] with an impact on the neurovascular unit and blood–brain barrier integrity [13]. Furthermore, obesity-induced neuroinflammation is linked to behavioral changes in the offspring such as autism and hyperactivity [14, 15]. We have previously reported that perinatal inflammation in the neonate results in hippocampal synaptopathology, autistic-like behaviors [16] and disruption of the blood–brain barrier in the hippocampus of mice [17].

A family of protein-tyrosine kinases, ephrins and their respective Eph receptors, are traditionally known as cell–cell interaction proteins. However, ephrins are also known to be important in metabolic regulation [18, 19] and have been shown to contribute to the suppression of adipose inflammatory responses. Thus, it has been suggested that a reduction in adipose ephrin signaling may accelerate adipose tissue inflammation in obesity [20]. The existing body of research on ephrin signaling also suggests that astrocytic ephrin-A3 signaling, mediated by EphA4 receptors, is necessary for controlling the abundance of glial glutamate transporters and glutamatergic synaptic plasticity in the brain [21, 22]. EphA4/ephrin-A3 cross-talk between astrocytes and pyramidal neurons is vital for stable dendritic spine organization during synaptic remodeling. EphA4 activation by ephrin-A3 induces spine retraction, while inhibiting EphA4/ephrin-A3 interactions distorts spine shape in hippocampal slices [23]. EphA4 forward signaling mediates the retraction of dendritic spines and reduces their number and size by remodeling the actin cytoskeleton and modifying adhesion receptor properties [24]. However, the role of the Eph-ephrin system in metabolic diseases remains poorly understood. Specifically, very little is currently known about the role of Eph-ephrin signaling in brain development and in relation to maternal obesity.

In this study, we determined the impact of maternal obesity on the brain plasticity in adult offspring, with a particular focus on EphA4/ephrinA3-signaling, astroglia and microglia reactions and factors that may affect the vasculature, including gap junction protein connexin 43 (Cx43), vessel coverage of astrocytic end-feet (aquaporin-4, AQP4). In addition, protein expression of the iron carrier protein, lipocalin-2 (LCN2) was determined by immunohistochemistry as it has been shown to influence astrocyte activation [25].

## Materials and methods

### Animals

All experimental protocols were approved by the Institutional Review Board of the University of Colorado Anschutz Medical Campus. We used a well-established mouse model of maternal obesity that shows extensive similarities to the human condition, including elevated levels of maternal leptin, glucose intolerance, activation of placental insulin and mTOR signaling, increased placental nutrient transport, and fetal overgrowth [26–28]. In brief, 12-week-old C57BL/6J female mice were fed either a control diet (D12489; Research Diets, New Brunswick, NJ, USA) or an obesogenic diet (Western Diet D12089B; Research Diets) consisting of pellets containing 10% and 40% calories from fat, respectively, as described previously [29]. Female mice fed the obesogenic diet also had ad libitum access to 20% sucrose solution supplemented with micronutrients and vitamins and minerals. Females were fed the obesogenic diet until they had gained 25% in body weight and were then mated overnight with males on control diet.

Dams were allowed to deliver spontaneously, and litters were culled to equal size (*n* = 6–8). Dams were maintained on their respective diets throughout pregnancy and lactation. The offspring were fed a control diet after weaning at 3 weeks of age. At 6 months of age, 13 offspring (control *n* = 7, obesity *n* = 6) for molecular analysis and 12 offspring (*n* = 6 per group) for histological analysis were euthanized and brain tissue collected. Both the studied male (+ 11%) and female (+ 8%) offspring of obese dams were heavier than offspring of control dams, in agreement as reported previously [30, 31].

### Tissue processing for histological analysis

Perfusion-fixed (saline/4%PFA) brains were divided into 2 hemispheres and one hemisphere was randomly selected and placed in a 30% sucrose solution (Sigma Aldrich) for 72 h, followed by snap-freezing using isopentane (Sigma Aldrich). Brain hemispheres were cut into 40-µm sections with sagittal orientation on a cryostat (Leica, CM 3050 S). The first section was selected randomly with a section sampling fraction (SSF) of 1/8 based on a systematic sampling method [32, 33]. One set of sections was used for Nissl staining with 0.25% thionin solution (Sigma T3387, France) as described before [34]. The other sets were used for glial fibrillary acidic protein (GFAP, astrocyte marker), ionized calcium binding adaptor molecule 1 (Iba1) (microglia marker), CD31 (endothelial cell marker) and aquaporin 4 (AQP4, end-feet of astrocytes), connexin-43 (Cx43, gap junction protein), myelin basic protein (MBP, myelination in white matter) and lipocalin-2 (LCN2, iron carrier protein) immunohistochemistry staining.

### Tissue processing for molecular analysis

In animal studies, maternal obesity has been shown to create significant functional or structural changes to specific areas of the brain, such as prefrontal cortex (PFC) and hippocampus [35]. Therefore, the tissue from 2 brain regions (PFC and hippocampus) were collected after euthanasia of the animal by decapitation and subsequently snap frozen for the molecular analysis of EphA4/ephrinA3-signaling and synaptic proteins.

### Immunohistochemistry

Free-floating brain sections were rinsed in phosphate-buffered saline (PBS, Gibco Invitrogen, Waltham, MA, USA) for 10 min followed by incubation in target retrieval solution (Dako, Glostrup, Denmark) at 85 °C for 40 min. Subsequently, sections were washed for 2 × 10 min in PBS, followed by blocking endogenous peroxidases (3% H_2_O_2_ in PBS, Sigma Aldrich) for 10 min. Sections were washed in PBS containing 0.25% Triton-X-100 (PBS-T, Sigma Aldrich). Next, sections were incubated in polyclonal primary rabbit anti-GFAP (1:500, Dako), rabbit anti-Iba1 (1:1000, Sigma Aldrich), rabbit anti-CD31 (1:500, Abcam), polyclonal rabbit anti-aquaporin-4 (1:500, Boster Biological Technology, PB9475), polyclonal rabbit anti-connexin-43 (1:500, C6219, Sigma Aldrich), monoclonal mouse anti myelin basic protein (MBP) (1:1000 clone SMI-94, 1:1,000; BioLegend 836504) and rat monoclonal anti lipocalin-2 (1:500, 70287, Abcam) overnight at 4 °C. The next day, sections were washed in PBS-T and subsequently incubated in polyclonal secondary biotinylated goat-anti-rabbit, goat-anti-mouse and goat-anti-rat antibodies (1:250, Vector Laboratories, Olean, NY, USA) in PBS-T for 2 h in room temperature (RT). Sections were washed in PBS-T followed by incubation in ABC elite solution (1.5% solution A + 1.5% solution B in PBS, Vector Laboratories) for 1 h at RT. Subsequently, sections were washed in PBS-T for 2 × 10 min and immunolabeling was performed by using 3,3-diaminobenzidine solution (Acros Organics, Geel, Belgium). In the last step, sections were washed in distilled H_2_O and PBS, mounted on gelatin-coated slides, dehydrated in 95%, 99% alcohol, xylene and cover slipped.

### Quantification of hippocampal volume

**Fig. 1:**
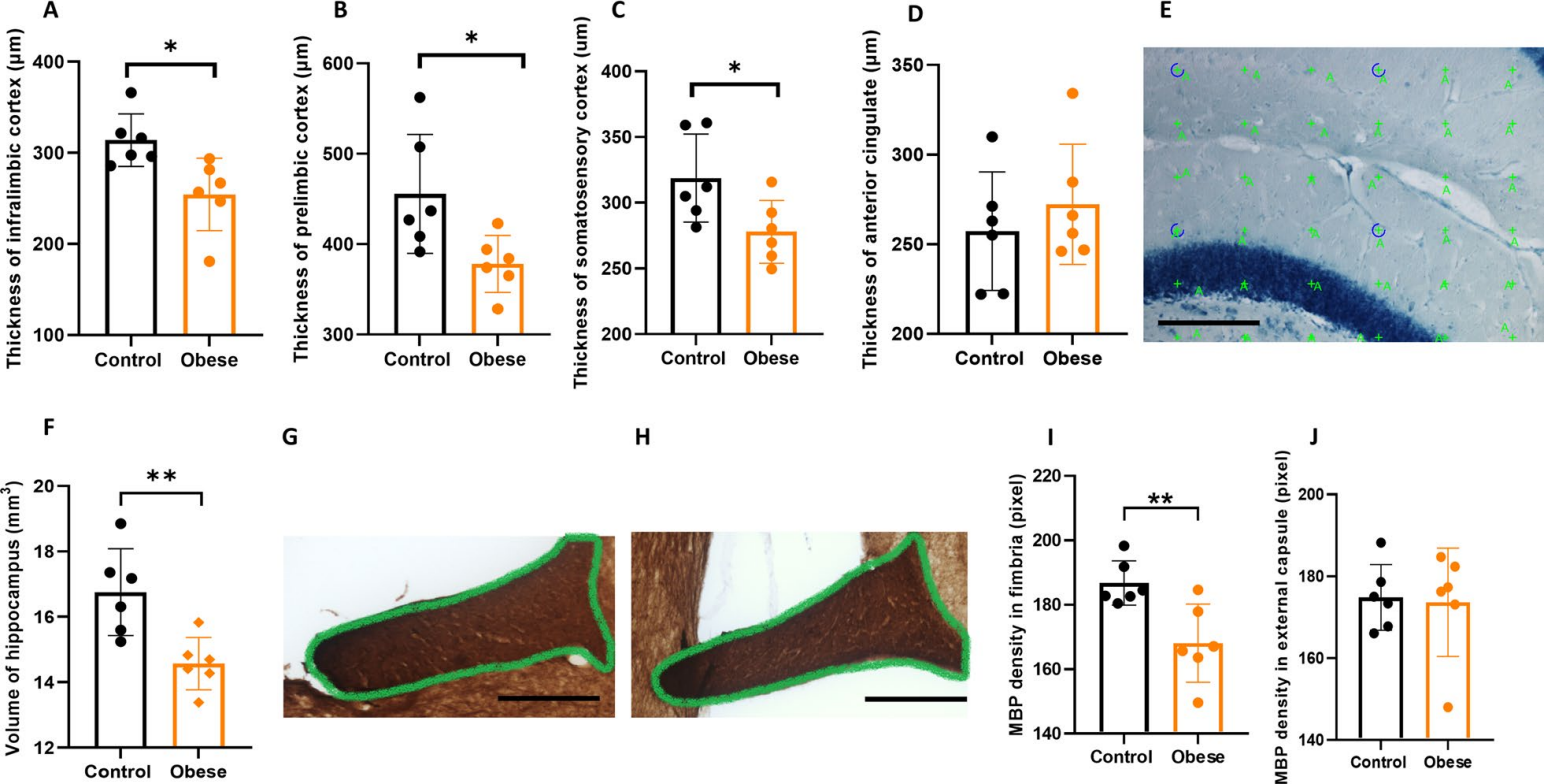
Effects of maternal obesity on the thickness of cortex, the volume of hippocampus and the white matter development in offspring: thickness of infralimbic cortex (**A**), prelimbic cortex (**B**) and somatosensory cortex (**C**) were significantly less in the offspring of obese dams without difference in the anterior cingulate cortex thickness (**D**); an example of Cavalieri point counting application on 40-μm-thick sagittal Nissl-stained section of the hippocampus for measuring the volume of hippocampus. Thirty-six green points hit the area and were counted. Scale bar = 200 μm (**E**); hippocampus was significantly smaller in the offspring of obese dams (**F**); examples of myelin basic protein (MBP) stained fimbria in the offspring from obese dams (delineated with green color) (**G**) and control group (delineated with green color). Scale bar = 400 µm (**H**); significant lower density of MBP was observed in hippocampal fimbria in offspring from obese dams (**I**); no difference in the MBP density in external capsule was observed between the groups (**J**). *p < 0.05, **p < 0.01, (*n* = 6/group)

The volume of the hippocampus was measured on Nissl-stained sections by Cavalieri estimator with point counting [36, 37] using a 10 × objective lens under light microscope modified for stereology with a digital camera (Leica DFC 295, Germany) and newCAST™ software (Visiopharm, Hørsholm, Denmark) (Fig. 1E). The formula used for determining the volume of the subregion was:

$$V= \Sigma P \cdot \left( \frac{a}{p} \right)\cdot T \cdot \frac{1}{{\text{SSF}}}$$

where *ΣP* is the total number of the points hitting hippocampus for each mouse, *(a/p)* is the area per test point; *T* is the section thickness (40-µm) and SSF is the section sampling fraction (1/8) [38].

### Measurement of cortical thickness

Cortical thickness was measured in clinically relevant brain regions (Nissl stained) including layer III of anterior cingulate, prelimbic, infralimbic and somatosensory cortex [39]. Three sections were used for each animal and two measurements were performed for each region on each section by systematic random sampling (6 measurements in total for each region per animal).

### Image acquisition, 3D reconstruction and morphological analysis of astrocytes and microglia

In order to study the effect of maternal obesity on the activity of hippocampal astrocytes and microglia, we analyzed several morphological features of astrocytes/microglia in the CA1 striatum radiatum (CA1.SR) subregion of hippocampus in the offspring. This study focuses on the CA1.SR subregion of the hippocampus for several key reasons. Firstly, this area is characterized by a high percentage (94.7%) of excitatory glutamatergic synapses, known for their notable synaptic plasticity [40]. The main focus of this study was to determine whether defects in ephrin-A3/EphA4-mediated cross-talk between inflammatory glia cells (astrocytes and microglia) and neurons in the hippocampus, which is critical in regulating synaptic plasticity, could explain some of the behavioral deficits following maternal obesity. Given that our investigations centered on assessing the impact of maternal obesity on the gliovascular unit in the offspring's hippocampus, we focused on CA1.SR subregion. Additionally, recent findings from our research have demonstrated that perinatal inflammation, identified as an early-life risk factor for impaired brain development, significantly influences the later-life development of the CA1.SR subregion of the hippocampus [38]. A total of 10 GFAP^+^/Iba1^+^ astrocytes/microglia were randomly selected for each animal for 3D reconstruction and morphological analysis based on the criteria: (1) cell bodies with clear border in the middle of the section thickness; (2) all branches are intact; (3) branches of the cell should be easily distinguishable from other cells or background staining. A systematic set of Z-stacks of images with a z-plane step size of 1 µm and selecting the middle of section as zero was obtained on GFAP/Iba1 stained sections using a 63 × oil-immersed objective lens on a light microscope modified for stereology. Applying this way of image acquisition gave us the possibility of image capturing of more than one astrocyte per image [38, 41]. The captured images were analyzed using Filament Tracers algorithm of the Imaris software (version 9.7; Bitplane AG, Zurich, Switzerland). Analyzed morphological parameters were: (1) number of cellular branches, (2) total length of the branches, and (3) Sholl analysis based on the radial distance from the center of the cell soma in 10-µm intervals for astrocytes and 5-µm intervals for microglia. In addition, to investigate details of the branching pattern of astrocytes, we did branch level analysis by considering 5 branching levels. Sphericity of astrocyte soma, as one of the indications of cell activation, was determined using Surface module of the Imaris software, as described previously [42]. Three-dimensional ellipsoid plots were generated by MATLAB (R2020b) according to: https://mathworld.wolfram.com [43].

### Estimation of number density of Iba1^+^ microglia and GFAP^+^ astrocytes in the CA1.SR subregion of hippocampus

Unbiased stereology measurements were performed to estimate the density of Iba1^+^ microglia (including ramified and amoeboid) and GFAP^+^ astrocytes in the CA1.SR subregion of the hippocampus. We applied a section sampling fraction (SSF) of 1/16 and area sampling fraction (ASF) = 30%, using 63 × oil-immersed lens and optical disector probe with the height of 15 µm (astrocytes) and 10 µm (microglia). The density of cells was calculated according to the following formula [38]:

$$N=\frac{\Sigma {Q}^{-}}{V}$$

where *N* is the number of cells per volume of brain region; *ΣQ–* is the number of counted cells; *V* is the volume of regions of interest on sampled sections.

### Cell soma volume measurement of astrocytes and microglia

The cell soma volume of astrocytes and microglia was quantified in the CA1.SR subregion of the hippocampus by applying 3D nucleator [41]. The mode was vertical uniform random (VUR) based on the assumption of rotational symmetry of astrocytes and microglia. Volume of cells was estimated with a 100 × oil-immersion objective lens. For each measurement, 50–80 cells per animal were analyzed with the optical disector set at a height of 15 μm.

### Measurement of the length density of CD31^+^, AQP4^+^ and LCN-2^+^ capillaries

We measured length density (L_v_) of CD31^+^ capillaries to discern whether changes in the length density of LCN-2^+^ or AQP4^+^ vessels could be attributed to alterations in capillaries themselves or to variations in the expression of LCN or AQP4 on the capillaries. The L_v_ of the CD31^+^, AQP4^+^ and LCN-2^+^ capillaries (based on LCN-2 expression in endothelial cells) were measured in the CA1.SR subregion of the hippocampus by applying the global spatial sampling method. This allows quantification of the length of the capillaries within a three-dimensional sampling box [44] using a 63 × oil immersion objective lens [45]. The measurements were carried out using computer-assisted stereological analysis of systematically sampled microscope fields by superimposing isotropic virtual planes in volume probes (the box corners of the probes).The following formula was used for measuring the capillaries' length density:

$${{\text{L}}}_{{\text{V}}}\left({\text{capillaries}} \right)=\frac{2\cdot p\left({\text{box}}\right)}{{\text{avg}} a\left({\text{plane}}\right)}\cdot \frac{\sum Q\left({\text{capillaries}} \right)}{\sum P}$$

where L_v_ (capillaries) is the length density of the capillaries which were positive for CD31, AQP4 and LCN-2; *ΣQ* (capillaries) is the sum of intersections between the test lines and the capillaries; *P* (box) is the number of box corners = 4; avg *a *(plane) is the average of the plane area; *ΣP* is the sum of the box corners hitting CA1.SR region.

### Measurement of hippocampal Cx43 expression

We captured images of three Cx43-stained hippocampal sections using a light microscope (5 × objective lens) modified for stereology. A histogram of the mean linear intensity using a 200-bin interval was generated in an automated procedure, which was independent of the background and threshold. The average intensity of 3 bands that measures the lightness of the image was then converted to a density for the darkness of the image (density = 255 − intensity) and compared between the groups [46].

### Measurement of the MBP density

Analysis of myelination in white matter was performed on three MBP stained sections per animal. The density of MBP was measured in two subregions of the brain, external capsule and fimbria, by performing the same method as described above.

### Reverse transcription-quantitative PCR

Total mRNA was isolated from the prefrontal cortex and hippocampus using the miRNeasy mini kit (Qiagen Inc.) according to the manufacturer’s recommendations. Total RNA concentration and purity were measured by a Nanodrop (Thermo Scientific) at 235, 260 and 280 nm. cDNA was prepared from 1ug RNA in a 20-μL reaction using QuantiTect Reverse Transcription Kit (Qiagen). Each PCR (20 μL), containing 2 μL cDNA (12 ng), 10 μL Quanti Fast SYBR Green PCR Master Mix (Qiagen) and 2 μL PCR primer (QuantiTech Primer Assay, Qiagen), was run on a LightCycler 480 (Roche, Sweden). The following primers were used: Efna3 QuantiTech Primer Assay (QT00320026), Epha4 QuantiTech Primer Assay (QT00093576), Dlg4 QuantiTech Primer Assay (QT00121695) and Syp QuantiTech Primer Assay (QT01042314), all from Qiagen. Melting curve analysis was performed to ensure that only one PCR product was obtained. For quantification and for estimation amplification efficiency, a standard curve was generated using increasing concentrations of cDNA. The amplified transcripts were quantified with the relative standard curve and normalized by the cDNA concentration using the Quant-IT OliGreen ssDNA Assay kit (Fisher Scientific).

### Statistical analysis

All data were analyzed using SPSS (IBM Corp. Released 2013, Version 26.0. Armonk, NY, United States). We used GraphPad Prism 8 (GraphPad Software Inc., USA) for generating plots. All analyses were performed blindly to the experimental conditions. Prior to performing statistical analysis, normal distribution of data was tested by making a Q–Q plot of the data. The variance homogeneity of data was examined by Levene’s test. We compared histological variables between two independent groups (Control and Obese) using independent t-test. For the molecular data, due to the low number of animals per group (*n* = 6–7) and high variability, non-parametric Mann–Whitney U test was used. We tested the correlation between variables by doing two tailed Pearson analysis (histological data) and Spearman analysis (molecular data). In all cases, the significance level was set at *p* < 0.05. The results are presented as mean ± standard deviation (SD).

## Results

### Smaller size of brain structures and reduced myelination in offspring of obese dams

By measuring the cortical thickness on Nissl-stained sections, we found that thickness of prelimbic, infralimbic and somatosensory cortex was significantly less in offspring of obese dams as compared to controls (*p* = 0.026, *p* = 0.014, *p* = 0.036). However, the thickness of anterior cingulate cortex was not different between the two groups (*p* = 0.453) (Fig. 1A–D). Measurement of the hippocampal volume on Nissl-stained sagittal sections showed significantly smaller size of hippocampus in offspring of obese dams as compared to controls (*p* = 0.006, Fig. 1E, F). By measuring the density of MBP staining in two white matter regions anatomically close to the hippocampus (external capsule and hippocampal fimbria), we found that MBP^+^ staining in the fimbria (Fig. 1G, I) was significantly lower in offspring of obese dams as compared to controls (*p* = 0.008), while there was no difference in the external capsule MBP + staining (*p* = 0.853) (Fig. 1H, J).

### Maternal obesity reduces the mRNA expression of EphA4 and synaptic protein PSD-95 in hippocampus, but not in the PFC of offspring

**Fig. 2:**
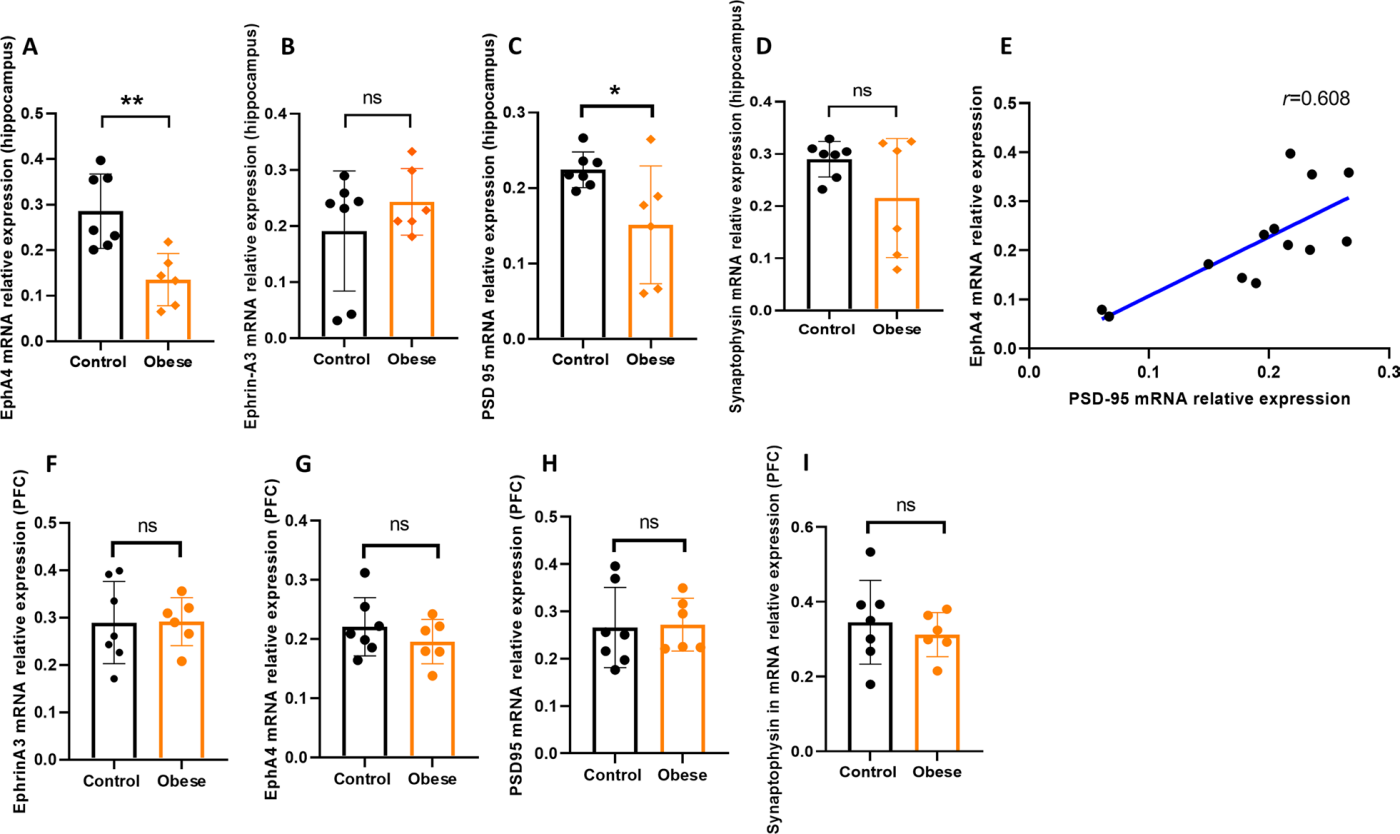
Effects of maternal obesity on mRNA expression of EphA4, ephrin-A3, PSD-95 and synaptophysin in the hippocampus and PFC of offspring: EphA4 and PSD-95 mRNA expressions significantly decreased in the hippocampus of offspring of obese dams (**A, C**); there was no difference in the hippocampal ephrin-A3 and synaptophysin mRNA levels between the groups (**B, D**); a significant correlation between EphA4 and PSD-95 mRNA expressions in the hippocampus was observed (**E**); there was no difference in the ephrin-A3, EphA4, PSD-95 and synaptophysin mRNA levels in the PFC between the groups (**F**–**I**). *p < 0.05, **p < 0.01, (*n* = 7/control group; *n* = 6/ obese group)

The mRNA level of EphA4 in the hippocampus of offspring of obese dams was significantly lower than levels in the control group (*p* = 0.005, Fig. 2A). However, there was no significant difference in the hippocampal ephrin-A3 mRNA level between the two groups (Fig. 2B). The mRNA level of PSD-95 was significantly lower in the hippocampus of offspring of obese dams as compared to offspring of control dams (*p* = 0.035, Fig. 2C) without differences in synaptophysin mRNA levels between groups (Fig. 2D). Moreover, the hippocampal mRNA expression of PSD-95 and EphA4 were positively correlated (*p* = 0.036, *r* = 0.608, Fig. 2E).

In contrast to the hippocampus, maternal obesity did not affect mRNA expression of EphA4, PSD-95 and synaptophysin in the PFC (*p* = 0.479; *p* = 0.329; *p* = 0.888; *p* = 0.535) (Fig. 2F–I). Thus, further analysis was performed in the hippocampus.

### Maternal obesity induces activation and decreases number density of astrocytes in the hippocampus of offspring

**Fig. 3:**
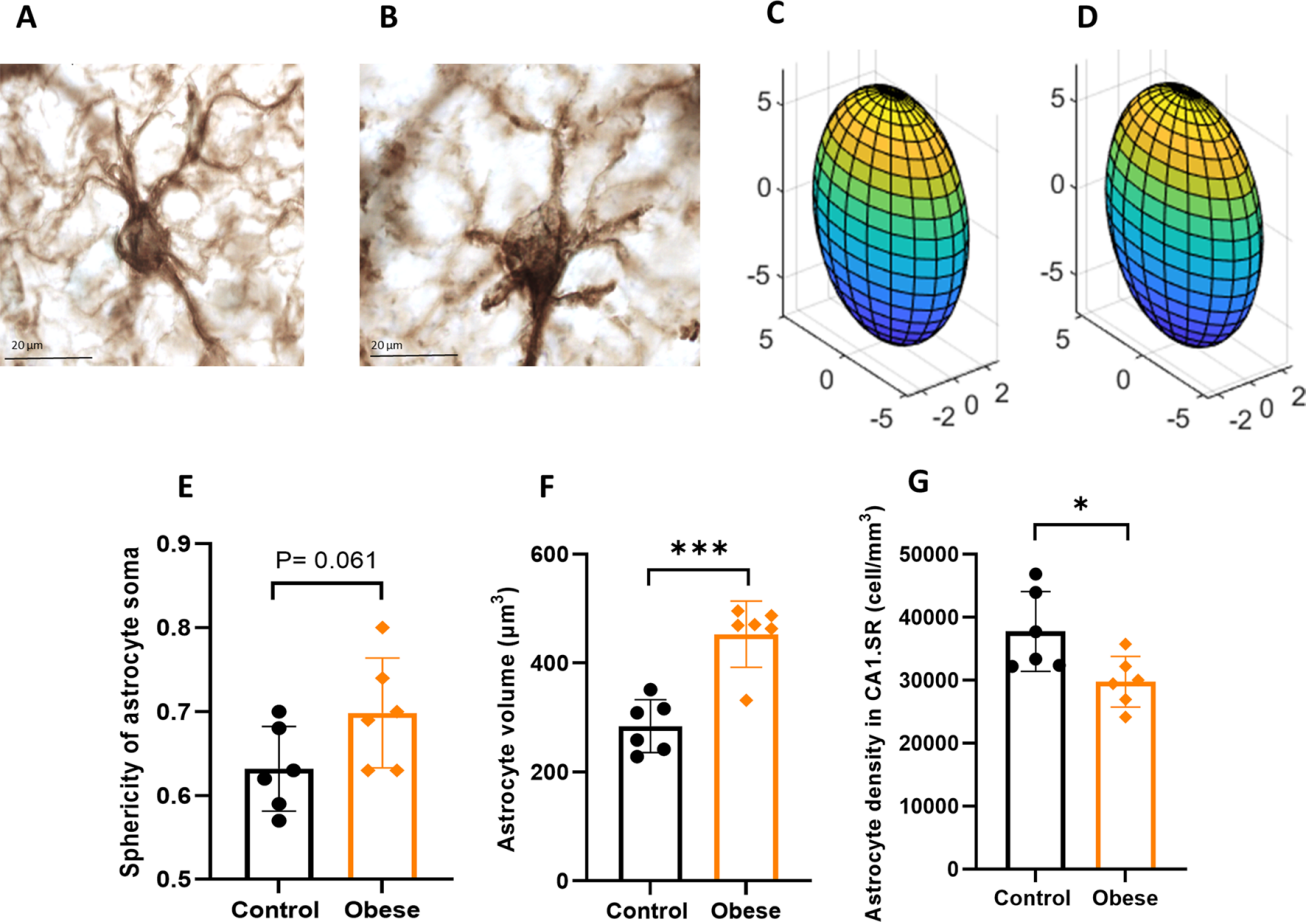
Effects of maternal obesity on sphericity, volume and number density of astrocytes in the offspring: examples of GFAP staining of astrocytes in the hippocampus of offspring of control dams (**A**) and offspring of obese dams (**B**), scale bar = 20 µm; three-dimensional plots of astrocyte ellipsoid shape in offspring of control dams (**C**) and offspring of obese dams (**D**); the effect of maternal obesity on offspring astrocyte spherical shape (**E**); larger size of astrocyte soma (hypertrophy) in the hippocampus of offspring of obese dams’ group (**F**); a significant lower density of astrocytes in CA1.SR subregion of hippocampus of offspring of obese dams (**G**); *p < 0.05, *** *p* < 0.001, (*n* = 6/group)

**Fig. 4:**
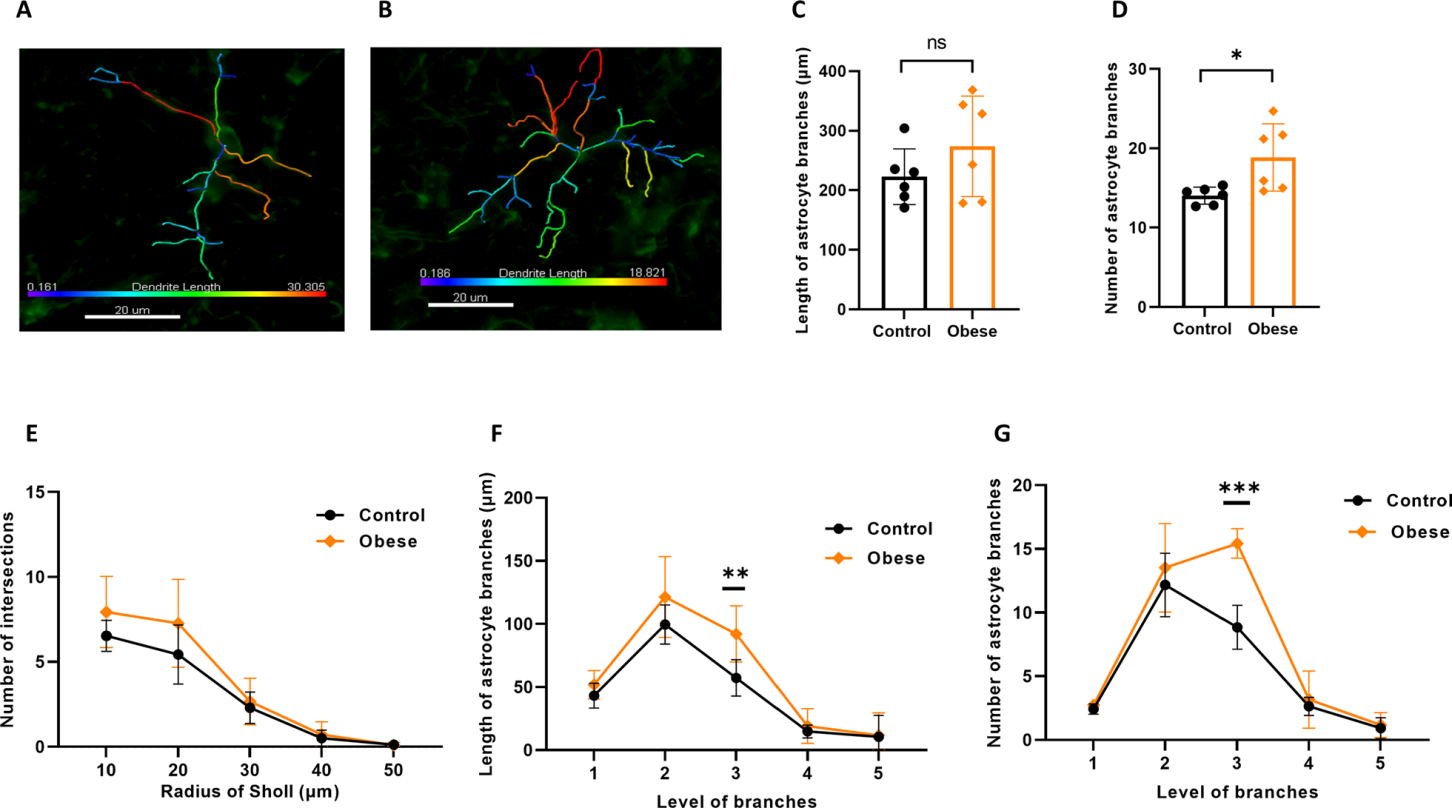
Effects of maternal obesity on astrocytes morphology in the hippocampus of offspring: examples of images of 3D reconstructed astrocyte in the CA1.SR subregion of hippocampus in offspring of control (**A**) and obese dams mice which includes more and longer branches (**B**); blue color indicates the shortest processes, and red color is an indicator of the longest astrocytic processes, scale bar = 20 μm; astrocyte morphology analysis indicated no significant difference in the length of astrocyte branches (**C**) but a significant higher number of branches (**D**). No difference in complexity of arborization of astrocytes branches (**E**) but an abnormal development of astrocyte branches in the hippocampus of offspring of obese dams as compared to offspring of control dams (**F, G**). *p < 0.05, **p < 0.01, ***p < 0.001, (*n* = 6/group)

GFAP-stained astrocytes in control (*p* = 0.000, Fig. 3A, F) group had significantly smaller soma than the obese group (Fig. 3A, B). To assess astrocyte activity, we did 3-D reconstruction of astrocytes and found a trend in increasing astrocyte soma (cell body) sphericity shape [42, 45] (*p* = 0.061, Fig. 3C–E) in offspring of obese dams as compared to offspring of control dams. Counting the number of GFAP^+^ astrocytes in the CA1.SR subregion of hippocampus showed that the number density of astrocytes was significantly lower in the offspring of obese dams (*p* = 0.026, Fig. 4G).

### Maternal obesity alters astrocyte morphology in the hippocampus of offspring

Astrocyte branching pattern was analyzed in 3D reconstructed astrocytes in the CA1.SR subregion of hippocampus in offspring of control (Fig. 4A) and obese dams (Fig. 4B). There was no difference in the length of astrocytic branches between obese and control groups (*p* = 0.231, Fig. 4C), but the number of astrocytic branches was significantly higher in the offspring of obese dams (*p* = 0.038, Fig. 4D). Sholl analysis indicated no significant difference in complexity of astrocyte branches between offspring of obese and control dams (Fig. 4E). To investigate the effect of maternal obesity on astrocyte branching growth, we did branch level analysis. At level three, astrocyte branches in the hippocampus were significantly longer (*p* = 0.009, Fig. 4F) and with a greater number (*p* = 0.000, Fig. 4G) in the offspring of obese dams as compared to offspring of control dams.

### Maternal obesity alters the morphology and the number of microglia in the hippocampus of offspring

**Fig. 5:**
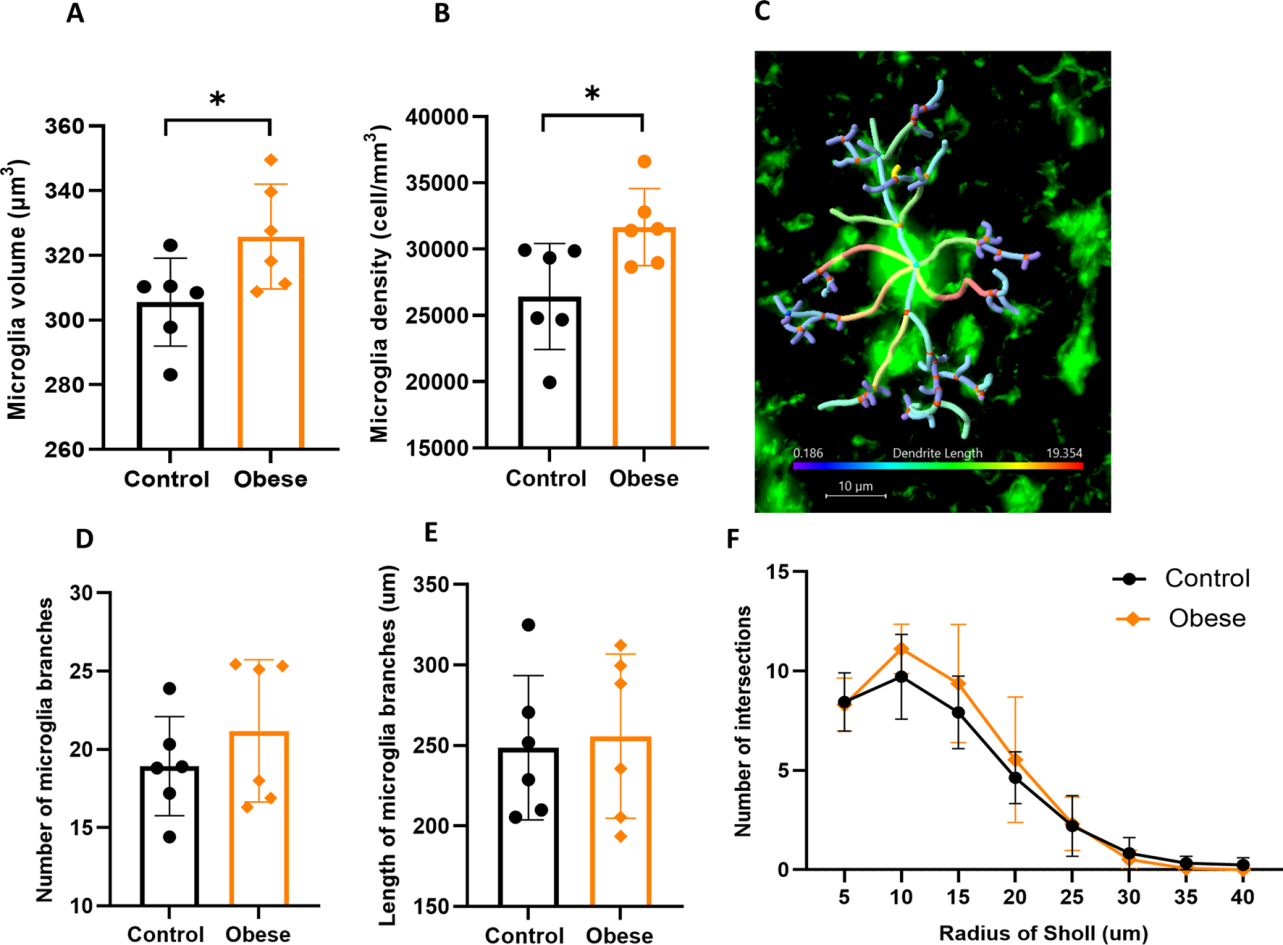
Effects of maternal obesity on the microglia morphology and density in the hippocampus of offspring: a significant bigger size of microglia soma (**A**); and increased density of microglia (**B**) were observed in the offspring of obese dams. *p < 0.05; example image of 3D reconstructed microglia in the CA1.SR subregion of hippocampus in offspring. Reconstructed microglia have different branch lengths from its starting points. Blue color indicates the shortest branches, and red color is an indicator of the longest microglia branches, scale bar = 10 μm (**C**); microglia morphology analysis indicated no significant difference in the number of microglia branches (**D**), length of microglia branches (**E**); and in complexity of arborization of microglia branches (**F**) (*n* = 6/group)

To assess activation of neuroinflammatory cells, the number, size of microglia soma and branching complexity were investigated. Measurement of microglia volume showed a significant larger soma in the offspring of obese dams (*p* = 0.041, Fig. 5A). Moreover, we found significantly higher microglia cell density in the offspring of obese dams as compared to offspring of control mice (*p* = 0.027, Fig. 5B). Interestingly, we found no significant difference in the branching complexity of microglia (length, number of branches and Sholl analysis) between two groups (*p* > 0.05) (Fig. 5C–F).

### Maternal obesity increases Cx43 gap junction expression in the hippocampus of offspring

**Fig. 6:**
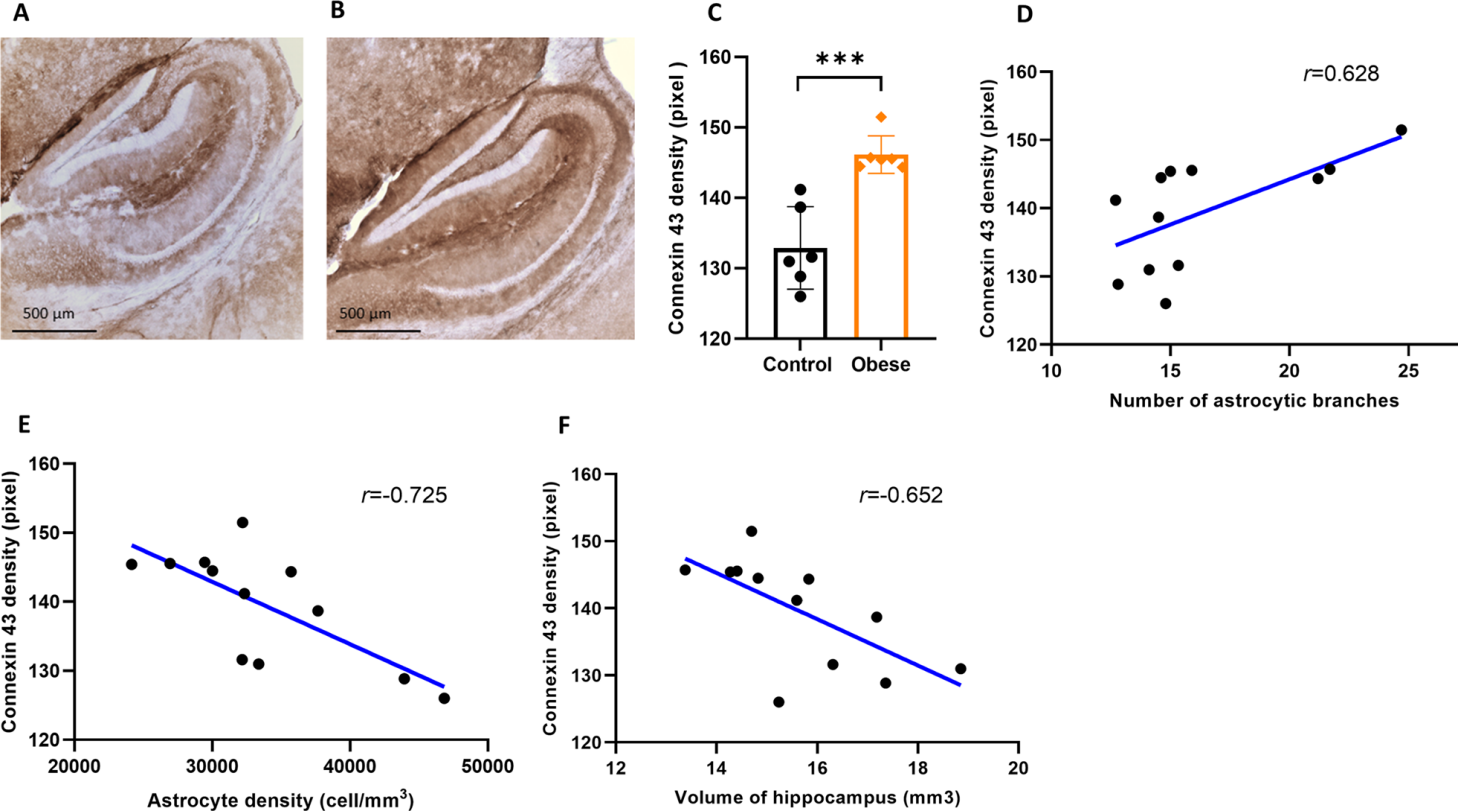
Effects of maternal obesity on the hippocampal Cx43 expression in offspring: examples of Cx43-stained hippocampal section from offspring of control (**A**) and obese dams (**B**), scale bar = 500 µm; a significant higher expression of Cx43 in the hippocampus of offspring of obese dams (**C**); a significant positive correlations between hippocampal Cx43 density and number of astrocytic branches (**D**); a significant negative correlation between Cx43 density, astrocyte density and volume of hippocampus (**E, F**), ***p < 0.001 (*n* = 6/group)

To investigate the impact of maternal obesity on the gap junctions between astrocytes, we measured Cx43 protein expression density on the hippocampal immunostained sections in offspring from control (Fig. 6A) and obese (Fig. 6B). The density of Cx43 protein expression was significantly higher in the hippocampus of offspring of obese dams as compared to offspring of controls dams (*p* = 0.000, Fig. 6C). Interestingly, the density of Cx43 expression was positively correlated with astrocytic number of branches (*p* = 0.029, *r* = 0.628, Fig. 6D), but was inversely correlated with the density of astrocytes (*p* = 0.008, *r* = − 0.725, Fig. 6E). In addition, hippocampal volume and Cx43 protein expression was negatively correlated (*p* = 0.021, *r* = − 0.652, Fig. 6F).

### Maternal obesity alters hippocampal capillary coverage by astrocyte end-feet and LCN

**Fig. 7:**
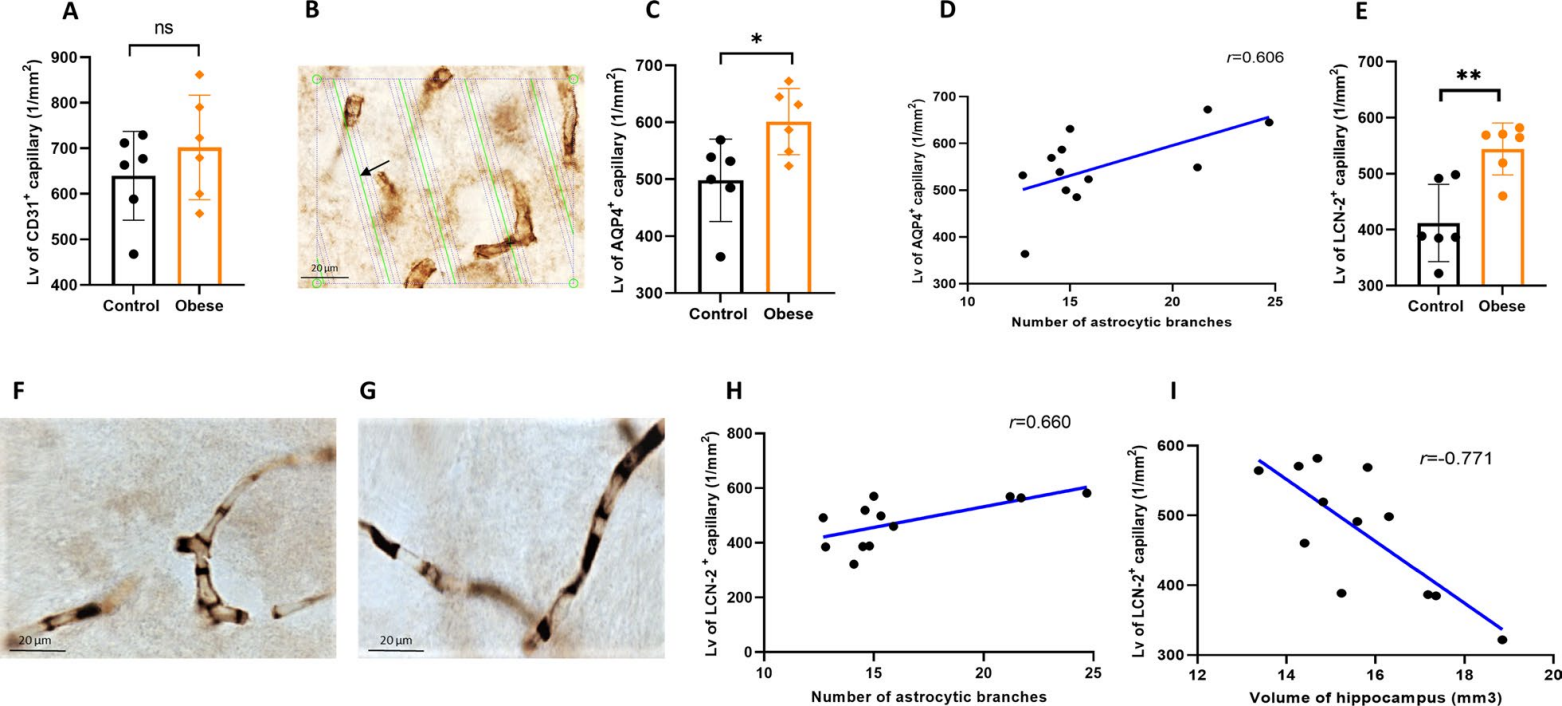
Effects of maternal obesity on the hippocampal vascularization and coverage of capillaries by end-feet of astrocytes in offspring: no significant difference in the length density of CD31^+^ capillaries between two groups of mice (**A**); an example of the length density of AQP4^+^ capillaries measurement. The green lines (arrow) represent the intersection between isotropic virtual plane and the focal plane. Capillaries that are in focus and intersected by virtual planes are counted as positive AQP4.For estimating the reference volume, we use the four corner points of the box.Scale bar = 20 μm (**B**); a significant higher coverage of capillaries with end-feet of astrocytes (**A**) without changes in vascularization in offspring of obese dams (**C**); a significant positive correlation between length density of AQP4^+^ capillaries and number of astrocytic branches (**D**); a significant higher endothelial expression of LCN2, suggesting longer LCN2^+^capillaries, in the hippocampus of offspring of obese dams (**E**); examples of hippocampal endothelial LCN2 expression in the offspring of control (**F**) and obese dams (**G**). Scale bar = 20 μm; a significant positive correlation between length density of LCN2^+^ capillaries and number of astrocytic branches (**H**); a significant negative correlation between length density of LCN2^+^ capillaries and volume of hippocampus (**I**). *p < 0.05, **p < 0.01, (*n* = 6/group)

We identified capillaries by immunohistochemical staining for CD31. The length density of CD31^+^ capillaries did not differ between groups *(p* = 0.335, Fig. 7A). Astrocytic end-feet were stained for AQP4 (Fig. 7B) and the results indicated significantly longer AQP4^+^ capillaries in the hippocampus of offspring of obese dams as compared to offspring of control mice (*p* = 0.021, Fig. 7C), likely reflecting a higher capillary coverage of astrocyte end-feet. Moreover, the length density of AQP4^+^ capillaries and the number of astrocyte branches were positively correlated (*p* = 0.037, *r* = 0.606, Fig. 7D). LCN-2 secretion in the brain under inflammatory conditions has been shown to promote morphological changes of astrocytes [25]. To measure LCN-2 immunohistochemical expression on the endothelial cells, we measured the length density of LCN-2^+^ capillaries in the hippocampus of offspring from control (Fig. 7F) and obese mothers (Fig. 7G). Our results showed significant upregulation of LCN-2 expression in the hippocampal endothelial cells in offspring of obese dams (*p* = 0.003) (Fig. 7E) which was significantly correlated with the number of astrocyte branches (*p* = 0.019, *r* = 0.660, Fig. 7H). Moreover, hippocampal volume and LCN-2 endothelial cell expression was negatively correlated (*p* = 0.003, *r* = − 0.771, Fig. 7I).

## Discussion

Maternal obesity is a risk factor for disrupted neurodevelopment [47]. In animal studies, maternal obesity induced by genetic alteration or dietary methods has been shown to lead to significant functional or structural changes to specific areas of the brain, such as PFC and hippocampus [35]. Eph/ephrin proteins regulate several neurodevelopmental processes such as migration of neurons, axon outgrowth, synaptic plasticity and angiogenesis [48]. To investigate the impact of maternal obesity on offspring brain development, we used a well-established mouse model of maternal obesity that shows extensive similarities to the human condition [26]. Our main findings are that maternal obesity was associated with smaller hippocampus and cerebral cortex, impaired myelination and reduced density of astrocytes, while astrocytes and microglia at some level displayed morphology consistent with activation. Astrocyte end-feet coverage of brain capillaries, gap junction protein Cx43 and LCN-2 were increased. At the same time, hippocampal mRNA expression of EphA4 and the post-synaptic protein PSD95 were decreased without significant change in the PFC. These results demonstrate that maternal obesity has an overall impact on brain size and specifically in the hippocampus alters synaptic plasticity and gliovascular factors in the offspring, changes that may be mediated through reduced EphA4 signaling.

To assess the impact of obesity on global brain development, we measured the tissue volume of the hippocampus, cortical white matter thickness and myelination in several brain regions. We found that offspring from obese mothers showed significant reductions in the volume of the hippocampus compared to offspring from lean mothers. These findings are in agreement with recent clinical studies reporting reduced hippocampal volume across various subregions of the hippocampus following maternal obesity [49]. Maternal obesity has also been shown to negatively influence frontal and parietal lobe white matter development in humans [39] and imaging studies show lower microstructure integrity of frontal white matter regions in neonates born to obese mothers compared to normal-weight mothers [50]. Similarly, we found that the thickness of the infralimbic, prelimbic and the somatosensory cortex was significantly decreased in offspring from obese mothers while the anterior cingulate cortex was not affected, indicating differential impact of maternal obesity on cortical development. Further, we found reduced myelination in offspring to obese mothers, which concurs with previous studies demonstrating decreased MBP staining in the cortex in male mice born to dams fed a high fat diet during pregnancy [51]. Taken together, our study and previously published data suggest that maternal obesity has widespread detrimental effects on structural development and myelination in the brain of the offspring.

Recent evidence indicates that EphA/ephrin-A signaling modulates cell processes such as adhesion/de-adhesion, apoptosis and migration, thereby influencing brain plasticity and size [52, 53]. Accordingly, impairment in brain EphA/ephrin-A signaling following exposure to obesity in pregnancy may be one of the possible mechanisms underlying smaller size of the hippocampus and impairment in white matter development in the offspring of obese mothers. We also found a significant positive correlation between EphA4 and PSD-95 expressions in the hippocampus. Our results indicate that maternal obesity does not affect EphA4/ephrin-A3 signaling or synaptic proteins in the PFC in the offspring. In contrast, we found decreased EphA4 gene expression together with deficit in PSD-95 in the hippocampus of 6-month-old offspring, without changes in epherin-A3 expression. The EphA4 receptor is mostly expressed on dendritic spines of neurons in the mouse hippocampus, while ephrin-A3 is localized to astrocytic processes surrounding the spines. The interaction between EphA4 and ephrin-A3 is important for synaptic plasticity in the hippocampus as long-term potentiation (LTP) at the CA3–CA1 synapse is modulated by EphA4 in the post-synaptic CA1 cell and by ephrin-A3, as a ligand of EphA4 on astrocytes [54]. EphA4 is strongly associated with PSD-95- post-synaptic density fractions [55] as EphA4 forward signaling mediates the retraction of dendritic spines and reduces their number and size by remodeling the actin cytoskeleton and modifying adhesion receptor properties [24]. Therefore, one of the possible explanations of observing no changes in the EphA4/ephrin-A3 signaling or synaptic proteins in the frontal cortex could be due to cell-type specific expression of EphA4 and ephrin-A3. Maternal obesity can lead to epigenetic modifications that influence gene expression. Changes in DNA methylation, histone modifications, or non-coding RNA expression might occur differentially between brain regions [56].

The effects of maternal obesity on gene expression may be time-sensitive and occur during critical periods of brain development in different brain regions. We are assuming changes in EphA4/ephrin-A3 expressions are more evident in the hippocampus since this region due to the high plasticity is particularly vulnerable to early life risk factors such as maternal obesity [57].

Therefore, our results suggest that maternal obesity has long-term influence on synaptic plasticity in the hippocampus and impairment of EphA4 signaling is one of the possible mechanisms underlying the abnormal synaptic plasticity.

As activation of EphA4 is one of the triggers for astrogliosis [58] and based on our finding of lower EphA4 expression in the hippocampus, we assessed the impact of maternal obesity on astrogliosis and astrocyte activation by determining astrocyte morphology and cell numbers in the CA1.SR subregion of the hippocampus. We found that while there was an increase in astrocyte cell soma size, indicating activation [42], the density of astrocytes was reduced in the obese group compared to control animals. In our study, we found a significant down regulation of EphA4 in the hippocampus which could be the possible explanation for lower density of astrocytes. Similarly, reduced parenchymal GFAP expression was reported in 16-month-old offspring following maternal high fat diet [13]. Astrocyte activity is a complex and multifaceted phenomenon. So, our results indicated trend into the sphericity of astrocyte soma in the hippocampus of offspring from obese dams. Soma sphericity, primarily relates to the shape of the astrocyte cell body. Changes in soma shape can be influenced by factors such as cell swelling which can be as a part of astrocyte activity. Therefore, astrocyte morphology, including soma shape, can provide additional context, but is not a direct indicator of activity and that is a reason we considered multiple parameters when studied astrocyte activity to gain a comprehensive understanding of alteration in astrocytes activity based on the morphology. These data suggest that astrocyte function may be affected in offspring following maternal obesity. Cx43 is the major gap junction protein in the hippocampus and is critical for the maintenance of normal shape and function of astrocytes [59]. It has been shown that overexpression of Cx43 in astrocytes restores neurite growth following oxygen glucose deprivation and reperfusion in mixed cultures, suggesting beneficial effects of high Cx43 levels [60]. In our study, we found a significant increase in Cx43 expression in the hippocampus of offspring of obese mice compared to the control mice and Cx43 expression was negatively correlated with the density of astrocytes in the hippocampus and with hippocampal volume. Therefore, we speculate that the lower expression of EphA4 and lower density of astrocytes may reflect a feed-forward cycle, which could be initiated by an abnormal distribution of Cx43. Indeed, maternal obesity may alter the homeostasis in astrocytes, disrupting the neuron–glia communication, by influencing the dynamic distribution of Cx43 [60–62].

The expression of Cx43 is not limited to astrocytes, but is also present on vascular endothelial cells, specifically at branch points of vessels, and has an important role in gliovascular cross-talk [63, 64]. Thus, we explored the possibility that altered Cx43 expression was related to hippocampal vascular changes. We found no significant difference in hippocampal vascularization between groups, which is similar to findings in adult offspring following pre- and post-natal high fat feeding [13]. On the other hand, astrocyte end-feet coverage of capillaries, as shown by AQP4 staining, was increased in the obese group. It has been suggested that Cx43 can affect organization of astrocyte end-feet [65] and Cx43 has been implicated in regulation of vascular permeability [66]. Further study will be necessary to understand the connection between changes in Cx43 and astrocytic AQP4 expression following maternal obesity and whether such changes have functional effects on for example blood–brain barrier permeability or changes in vascular flow.

Maternal obesity in rats showed increased expression of inflammatory markers including increased number of microglia cells in the hippocampus of offspring after birth [67, 68]. Similarly, we found that both volume and number of microglia were significantly increased in offspring of obese mothers compared to control animals. Concerning the microglia's branching pattern, we observed hyper-ramified microglia exhibiting elongation of their processes and heightened branch complexity, as opposed to adopting an amoeboid shape, which typically indicates early activation stages. This phenomenon is believed to represent an intermediate phase in microglial activation or a mild response to stimuli [69]. Considering that Eph/ephrin signaling has been reported to promote microglial polarization [70], changes in the EphA4 expression in the hippocampus may be associated with microglia activation. However, further study is needed to explore details of the impact of maternal obesity on microglia phenotype. It is also unclear how maternal obesity mediates signals to promote microgliosis.

In this study, we observed significant increase in capillaries’ LCN-2 expression in the offspring from obese dams and that is suggesting the effect of diet during pregnancy on endothelial production of LCN-2. LCN-2 is also known as neutrophil gelatinase-associated lipocalin (NGAL), siderocalin, which regulates various inflammatory processes in the CNS [71] by contributing to the activation of glia cells and promoting neurotoxicity [72]. We showed that increased LCN-2 was linked to cerebrovascular alteration following neonatal infection in mice and to blood C-reactive protein levels in preterm infants [73]. It has also been reported that LCN-2 can be neurotoxic in chronic, non-infectious inflammatory conditions [74]. Moreover, greater production of LCN2 in obesity has been observed by transcriptional regulation of the Lcn2 gene, NF-kappaB and CCAAT/enhancer-binding protein (C/EBP) pathways. Interestingly, Olson et al. showed that chronic inflammation resulted in increased secretion of LCN-2 from endothelial cells, which resulted in hippocampal microglia activation, neuronal dysfunction and impairment of spatial reference memory [75]. Therefore, the elevated endothelial LCN-2 expression in the brain of the offspring of obese dams may represent one link between maternal obesity and disrupted neurodevelopment in the adult offspring. Cytosolic LCN-2 within reactive astrocytes has been demonstrated to play a crucial role in the astrocytic uptake of myelin, potentially contributing significantly to the process of demyelination. Notably, hypertrophic astrocytes exhibit the ability to phagocytose myelin post-cortical ischemia, thereby promoting demyelination through the LCN-2/LRP1 pathway [76]. Our investigation also revealed a noteworthy positive correlation between the length density of LCN-2^+^ capillaries and the branching number of astrocytes, accompanied by abnormal myelination in the hippocampal fimbria following maternal obesity. Therefore, further study is needed to comprehensively grasp the impact of maternal obesity on the astrocytic LCN-2/LRP1 pathway.

## Limitations

Our study investigated the impact of maternal obesity on hippocampal glio-vascular cross-talk by assessing vascular morphology and glia cells in association with Eph/ephrin signaling. It remains to be determined if the reported changes are also associated with functional deficits. Therefore, future work should investigate the functional implications of altered hippocampal gliovascular unit in connection with ephrin signaling by examining whether these alterations are associated with cognitive or behavioral impairments in offspring of maternal obesity. Further, the effect of maternal obesity on brain development and ephrin signaling may differ between sexes, which should be further studied.

## Conclusion

Maternal obesity alters the development of the gliovascular compartment and decreases the mRNA expression of EphA4 and PSD-95 in the hippocampus of adult offspring. These changes may change synaptic plasticity and link maternal obesity to neurodevelopmental/neuropsychiatric disorders in the offspring.

## Data Availability

Raw data were generated at the University of Gothenburg, Institute of Neuroscience and Physiology. Derived data supporting the findings of this study are available from the corresponding author on request.
